# MARCKSL1–2 reverses docetaxel-resistance of lung adenocarcinoma cells by recruiting SUZ12 to suppress HDAC1 and elevate miR-200b

**DOI:** 10.1186/s12943-022-01605-w

**Published:** 2022-07-21

**Authors:** Min Jiang, Feng Qi, Kai Zhang, Xiaofei Zhang, Jingjing Ma, Suhua Xia, Longbang Chen, Zhengyuan Yu, Jing Chen, Dongqin Chen

**Affiliations:** 1 Department of Medical Oncology, The First Affiliated Hospital of Soochow University, No.188 Shizi Street, Gusu District, Suzhou, 215006 Jiangsu China; 2 Department of Pharmacy, The Fourth Affiliated Hospital of Nantong University, Yancheng, 224005 Jiangsu China; 3 Department of Respiratory Medicine, Nanjing First Hospital, Nanjing Medical University, Nanjing, 210006 Jiangsu China; 4 Department of Medical Oncology, Ren Ji Hospital, Shanghai Jiao Tong University School of Medicine, No.160 Pujian Road, Pudong New District, Shanghai, 200127 China; 5 Department of Pharmacy, The First Affiliated Hospital of Soochow University, No.188 Shizi Street, Gusu District, Suzhou, 215006 Jiangsu China; 6 Department of Medical Oncology, Nanjing Jinling Hospital, School of Medicine, Nanjing University, Nanjing, 210008 Jiangsu China; 7 Department of Biochemistry and Molecular Biology, School of Medicine & Holistic Integrative Medicine, Nanjing University of Chinese Medicine, No.138 Xianlin Avenue, Nanjing, 210023 Jiangsu China; 8 Department of Medical Oncology, Baoshan Branch, Ren Ji Hospital, Shanghai Jiao Tong University School of Medicine, No.1058 Huanzhen North Road, Baoshan District, Shanghai, 200444 China; 9 Department of Medical Oncology, The Affiliated Cancer Hospital of Nanjing Medical University & Jiangsu Cancer Hospital & Jiangsu Institute of Cancer Research, No.42 Baiziting Road, Xuanwu District, Nanjing, 210009 Jiangsu China

**Keywords:** Lung adenocarcinoma, MARCKSL1–2, HDAC1, miR-200b, Docetaxel-resistance, SUZ12

## Abstract

**Background:**

Long non-coding RNAs (lncRNAs) are implicated in the development of multiple cancers. In our previous study, we demonstrated that HDAC1/4-mediated silencing of microRNA-200b (miR-200b) enhances docetaxel (DTX)-resistance of human lung adenocarcinoma (LAD) cells.

**Methods and results:**

Herein, we probed the function of LncRNA MARCKSL1–2 (MARCKSL1-transcript variant 2, NR_052852.1) in DTX resistance of LAD cells. It was found that MARCKSL1–2 expression was markedly reduced in DTX-resistant LAD cells. Through gain- or loss- of function assays, colony formation assay, EdU assay, TUNEL assay, and flow cytometry analysis, we found that MARCKSL1–2 suppressed the growth and DTX resistance of both parental and DTX-resistant LAD cells. Moreover, we found that MARCKSL1–2 functioned in LAD through increasing miR-200b expression and repressing HDAC1. Mechanistically, MARCKSL1–2 recruited the suppressor of zeste 12 (SUZ12) to the promoter of histone deacetylase 1 (HDAC1) to strengthen histone H3 lysine 27 trimethylation (H3K27me3) of HDAC1 promoter, thereby reducing HDAC1 expression. MARCKSL1–2 up-regulated miR-200b by blocking the suppressive effect of HDAC1 on the histone acetylation modification at miR-200b promoter. Furthermore, in vivo analysis using mouse xenograft tumor model supported that overexpression of MARCKSL1–2 attenuated the DTX resistance in LAD tumors.

**Conclusions:**

We confirmed that MARCKSL1–2 alleviated DTX resistance in LAD cells by abolishing the inhibitory effect of HDAC1 on miR-200b via the recruitment of SUZ12. MARCKSL1–2 could be a promising target to improve the chemotherapy of LAD.

**Graphical abstract:**

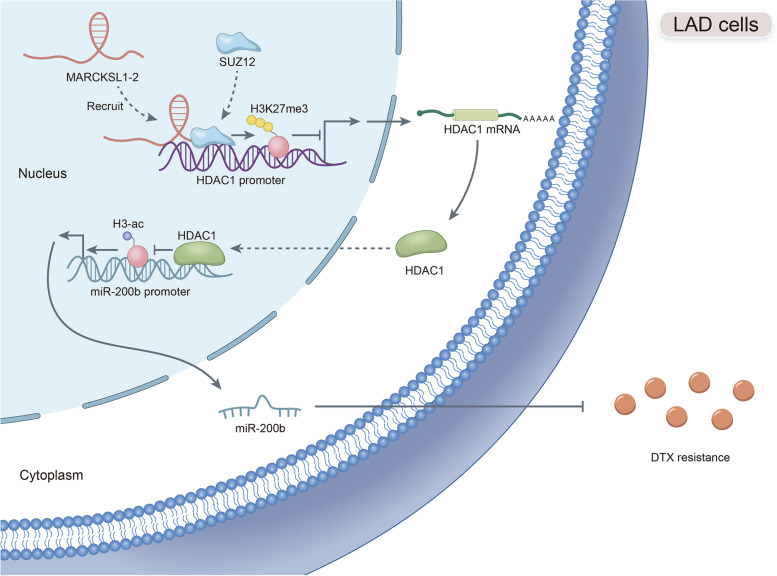

**Supplementary Information:**

The online version contains supplementary material available at 10.1186/s12943-022-01605-w.

## Background

Lung cancer is one of the most common malignant tumors and the main causes of cancer-related death worldwide [1, 2]. Non-small cell lung cancer (NSCLC) accounts for about 85% of all lung cancer cases. Lung adenocarcinoma (LAD) is the most frequent type of NSCLC [3]. Despite great progress in the development of chemotherapies, chemoresistance remains an obstacle for cancer treatment. Docetaxel (DTX) is an effective drug for the treatment of various cancers including LAD [4] However, tumor recurrence occurs in LAD patients with DTX treatment due to DTX resistance [5]. Hence, the potential mechanisms underlying DTX resistance in LAD need to be explored.

Recent studies suggest that over 98% of the human genome has no protein-coding function [6]. Long non-coding RNAs (lncRNAs) belong to a kind of RNAs with more than 200 nucleotides in length [7], and their abnormal expression is closely related to tumor progression, apoptosis, metastasis, and chemoresistance [8]. Moreover, lncRNAs are implicated in the pathogenesis of human diseases through regulating gene expression via their controls in chromatin modification, gene transcription, and post-transcriptional modification [9, 10]. Previous studies have demonstrated that lncRNAs are key regulators of LAD chemoresistance [11]. Xin Tian et al. found that knockdown of lncRNA ENST00000500843 enhances the resistance of LAD cells to paclitaxel [12]. Moreover, XIST is responsible for cisplatin resistance in LAD cells via the let-7i/BAG-1 axis [13]. NR_052852.1 (MARCKSL1-transcript variant 2, LncRNA MARCKSL1–2) is a novel lncRNA that has not been documented in LAD. Here, we investigated the role of MARCKSL1–2 in DTX resistance in LAD.

It was reported that HDAC1/4-mediated miR-200b knockdown enhances DTX resistance in human LAD cells [14]. The participation of miR-200b in cancer progression has been validated. For example, lncRNA ZFAS1 promotes the growth of osteosarcoma cells by targeting miR-200b and miR-200c to up-regulate BMI1 [15]. MiR-200b affects cell proliferation and migration by directly regulating ZEB2 in gastric carcinoma [16]. In the meantime, reports have revealed that histone deacetylase 1 (HDAC1) plays vital roles in multiple cancers, such as laryngeal squamous cell carcinoma [17], ovarian cancer [18], and NSCLC [19]. In this study, we evaluated the relationship among MARCKSL1–2, miR-200b, and HDAC1 in human LAD cells, as well as their association with DTX resistance.

## Materials and methods

### Patients

Tissues were gathered from 60 patients with advanced LAD and 20 patients with other lung diseases (control group) in the Nanjing General Hospital of Nanjing Military Command between March 2005 and January 2010. All 60 patients with LAD met the previously described criteria [20]. Tumor response to chemotherapy was assessed via computed tomography (CT) every two or three cycles of chemotherapy, and defined according to the Response Evaluation Criteria in Solid Tumors as follows: progressive disease (PD), stable disease (SD), complete response (CR), or partial response (PR) [20]. PD and SD were regarded as “insensitive”, whereas CR and PR were considered as “sensitive” [20]. Tissues were rapidly frozen with liquid nitrogen and then stored at − 80 °C. Tissue acquisition was permitted by the Review Board of Hospital Ethics Committee of Nanjing General Hospital of Nanjing Military Command (Nanjing General Hospital of Nanjing Military Command, Nanjing University, China). All patients or authorized persons provided written informed consents.

### Cell culture and treatment

Human LAD H1299 cells were obtained from the ATCC Cell Bank (Manassas, VA, USA), and human LAD SPC-A1 cells were from Procell life Science & Technology Co., Ltd. (Wuhan, China). Cells were cultured in RPMI-1640 medium (Gibco, Rockville, MD, USA) supplemented with 1% penicillin/streptavidin (Gibco) and 10% FBS (Gibco). DTX-resistant H1299 and SPC-A1 cells, termed H1299/DTX and SPC-A1/DTX, respectively, were established in our laboratory as described previously [14, 21]. H1299/DTX and SPC-A1/DTX cells were maintained in 50 μg/L of DTX (Selleck Chemicals, Houston, TX) and routinely grown as described in a previous work [22]. All cells were maintained in a humidified incubator with 5% CO_2_ at 37 °C.

### Quantitative real-time polymerase chain reaction (RT-qPCR)

Total RNA extraction was performed with TRIzol reagent (Takara, Shiga, Japan), and then cDNA was synthesized by using PrimeScript RT Reagent Kit (Takara) as per the manufacturer’s instruction. Quantitative analyses were performed using SYBR PrimeScript RT-qPCR Kit (Takara). Relative gene expression was detected based on 2^-ΔΔCt^ method by normalizing to GAPDH mRNA or U6 snRNA.

### Subcellular fractionation

PARIS™ Kit (Ambion, Austin, TX) was used for the nuclear/cytoplasmic fractionation in line with the manufacturer’s instruction. In brief, cells (1 × 10^7^) were collected and suspended in cell fractionation buffer and then lysed in cell disruption buffer. The MARCKSL1–2 content of both cell fractions was examined by RT-qPCR.

### Fluorescent in situ hybridization (FISH)

RNA FISH probe for MARCKSL1–2 was designed by Ribobio Company (Guangzhou, China). After culturing with probes in hybridization solution, cell samples were counter-stained in DAPI solution and then visualized using a fluorescence microscope (Olympus Corp., Tokyo, Japan).

### Cell transfection

The specific shRNAs targeting MARCKSL1–2 (shMARCKSL1–2#1 and shMARCKSL1–2#2), HDAC1 (sh/HDAC1), or SUZ12 (shSUZ12#1 and shSUZ12#2), as well as relative control shRNAs (sh/Ctrl), were procured from GenePharma (Shanghai, China). Then, the above shRNAs were cloned into pLKO.1 shRNA lentiviral vector (Addgene, Shanghai, China), followed by co-transfection of psPAX2 lentiviral packaging vector (Addgene) into HEK-293 T cells. Next, the collected lentiviruses particles containing the above respective shRNAs were used to infect LAD cells, and the stable cells were selected by puromycin treatment. For the overexpression of MARCKSL1–2, HDAC1, or SUZ12, the respective cDNA sequences were separately sub-cloned into pcDNA3.1 vectors (Invitrogen, Carlsbad, CA, USA) to construct pcDNA/MARCKSL1–2, pcDNA/HDAC1, and pcDNA/SUZ12 vectors. The empty vector (pcDNA3.1) was used as the negative control. Besides, cells were transfected with miR-200b mimics/inhibitor (Ribobio) to mimic or inhibit miR-200b, with NC mimics/inhibitor (Ribobio) as the negative control, respectively. The transfection of indicated pcDNA3.1 plasmid and miR mimics/inhibitor into LAD cells were achieved by using Lipofectamine 3000 (Invitrogen).

### Colony formation assay

Cells were seeded into 6-well plates and cultured for 2 weeks. Then, cells were fixed by formaldehyde for 30 min and stained by 0.5% crystal violet. Subsequently, the plates were imaged and the colonies with over 50 cells were counted manually.

### 5-Ethynyl-2′-deoxyuridine (EdU) assay

EdU assay was undertaken with BeyoClick™ EdU Cell Proliferation Kit with Alexa Fluor 594 (Beyotime, Shanghai, China). After washing in PBS, EdU solution was used to incubate cells for 2 h. Cell nuclei were then stained by DAPI solution. After washing, samples were observed with an inverted microscope (Olympus).

### Flow cytometry analysis

Cell apoptosis was assessed with flow cytometry (BD Biosciences, Franklin Lakes, NJ) as instructed. The Annexin V-FITC/PI Apoptosis Detection Kit (Elabscience, Wuhan, China) was utilized to stain cells for 15 min. Next, cell samples were collected from 6-well plates by centrifugation and the apoptotic cells were analyzed by flow cytometry.

### Terminal-deoxynucleoitidyl transferase mediated Nick end labeling (TUNEL) assay

Cells were rinsed using PBS and then fixed with ethanol. The apoptotic cells were stained by using TUNEL reagents (Merck KGaA, Darmstadt, Germany) in accordance with the standard method. The apoptotic cells were captured by an optical microscope (Olympus).

### Luciferase reporter assay

The promoter sequence of miR-200b or HDAC1 was separately inserted into the pGL3-basic vector (Promega, Madison, WI, USA) for luciferase reporter assays. Then, LAD cells (H1299 and SPC-A1) or DTX-resistant LAD cells (H1299/DTX and SPC-A1/DTX) were separately co-transfected with MARCKSL1–2 silencing or overexpression plasmids along with the recombinant pGL3 vectors for 48 h. The relative luciferase intensity was detected via luciferase reporter assay system (Promega).

### Chemosensitivity assay

Drug sensitivity was evaluated by cell counting kit-8 (CCK-8) assay. The parental LAD cells (H1299 and SPC-A1) and DTX-resistant LAD cells (H1299/DTX and SPC-A1/DTX) were treated with different doses of DTX and fixed in 96-well plates. Then, CCK-8 solution (Dojindo, Osaka, Japan) was added. OD value was measured at the absorbance of 450 nm. The 50% inhibitory concentration (IC_50_) value of indicated LAD cells to DTX treatment was defined as the DTX dose which induces 50% cell death.

### Chromatin immunoprecipitation (ChIP)

ChIP assay was undertaken by using the EZ ChIP™ Chromatin Immunoprecipitation Kit (Millipore, Bedford, MA, USA) according to the manufacturer’s direction. In short, cells were fixed with 1% formaldehyde for 15 min and subjected to ultrasonic treatment for shearing DNA into fragments (500 bp). The immunoprecipitation was implemented by mixing DNA fragments with 30 μl of magnetic beads conjugating with HDAC1 (#34589, 1/50 dilution, Cell signaling technology, Boston, MA, USA), SUZ12 (#3737, 1/100 dilution, Cell signaling technology), acetyl-histone H3 (#8173, 1/100 dilution, Cell signaling technology) or tri-methyl-histone H3 (lys27) (H3K27me3, #9733, 1/50 dilution, Cell signaling technology), and those anti-IgG antibodies (Millipore) acted as the negative control. Finally, the precipitated DNA fragments were analyzed via qPCR and agarose gel electrophoresis (AGE).

### RNA pull-down assay

The RNA pull-down assay was conducted by using the Pierce Magnetic RNA-Protein Pull-Down Kit (Thermo Fisher Scientific, Waltham, MA, USA). In short, total protein extracts, obtained from cells via RIPA lysis buffer, were mixed with biotinylated probe for MARCKSL1–2 or MARCKSL1–2 antisense (AS). The pulled-down mixture was processed with SDS-PAGE and sliver staining, and the proteins were analyzed via mass spectrometry. Finally, the existence of interested protein in the pull-down complex was detected through Western bloting.

### RNA immunoprecipitation (RIP)

Magna RIP™ RNA-Binding Protein Immunoprecipitation Kit (Millipore) was used for RIP assay in accordance with the instructions. The processed cells were conjugated with anti-SUZ12 antibody (#3737, 1/100 dilution, Cell signaling technology) and control IgG antibody (Millipore) on magnetic beads. After digestion, the precipitated RNAs were extracted for RT-qPCR analysis.

### Western blot

Cells were lysed in RIPA lysis buffer. Total proteins were separated on 12% SDS-PAGE. Then, they were transferred onto PVDF membranes and blocked with 5% skimmed milk. The primary antibodies against loading control GAPDH (ab181602, 1/10000 dilution), HDAC1 (ab109411, 1/2000 dilution) and SUZ12 (ab12073, 1/1000 dilution) were used. Then, incubation with the HRP-labelled secondary antibodies (ab216773, 1/10000 dilution) was conducted. All antibodies were bought from Abcam (Cambridge, MA, USA) and used as recommended. All protein bands were finally measured after adding the ECL reagent (Bio-Rad, Hercules, CA, USA). The experiment was independently repeated three times, and the representative blots of three independent experiments were provided in the Figures.

### In vivo experiments

Nude mice (4–6 weeks old) used for in vivo experiments were obtained from the Nanjing General Hospital of Nanjing Military Command. The animal study was approved by the Review Board of Hospital Ethics Committee of Nanjing General Hospital of Nanjing Military Command (Nanjing General Hospital of Nanjing Military Command, Nanjing University, China). Animal models were established by subcutaneously injecting 2 × 10^6^ H1299/DTX cells transfected with pcDNA3.1 and pcDNA3.1/MARCKSL1–2 (*n* = 6 each group). Two weeks later, three mice from the above two groups were randomly selected and treated with DTX (1 mg/kg) through intraperitoneal injections. After 4 weeks, all mice were sacrificed. The primary tumors were resected and weighed, followed by embedded with paraffin and fixed with formalin.

### Statistical analysis

All experimental data were obtained from three bio-repeated experiments and shown as the mean ± standard deviation (S.D.). GraphPad Prism 7 (La Jolla, CA) was used for statistical analyses. Comparison among multiple groups was analyzed by one-way or two-way ANOVA, and that between two groups was analyzed using Student’s t test. Results were considered statistically significant when a *p*-value< 0.05.

## Results

### Down-regulation of lncRNA MARCKSL1–2 is associated with DTX-resistance and poor prognosis in LAD patients

**Fig. 1:**
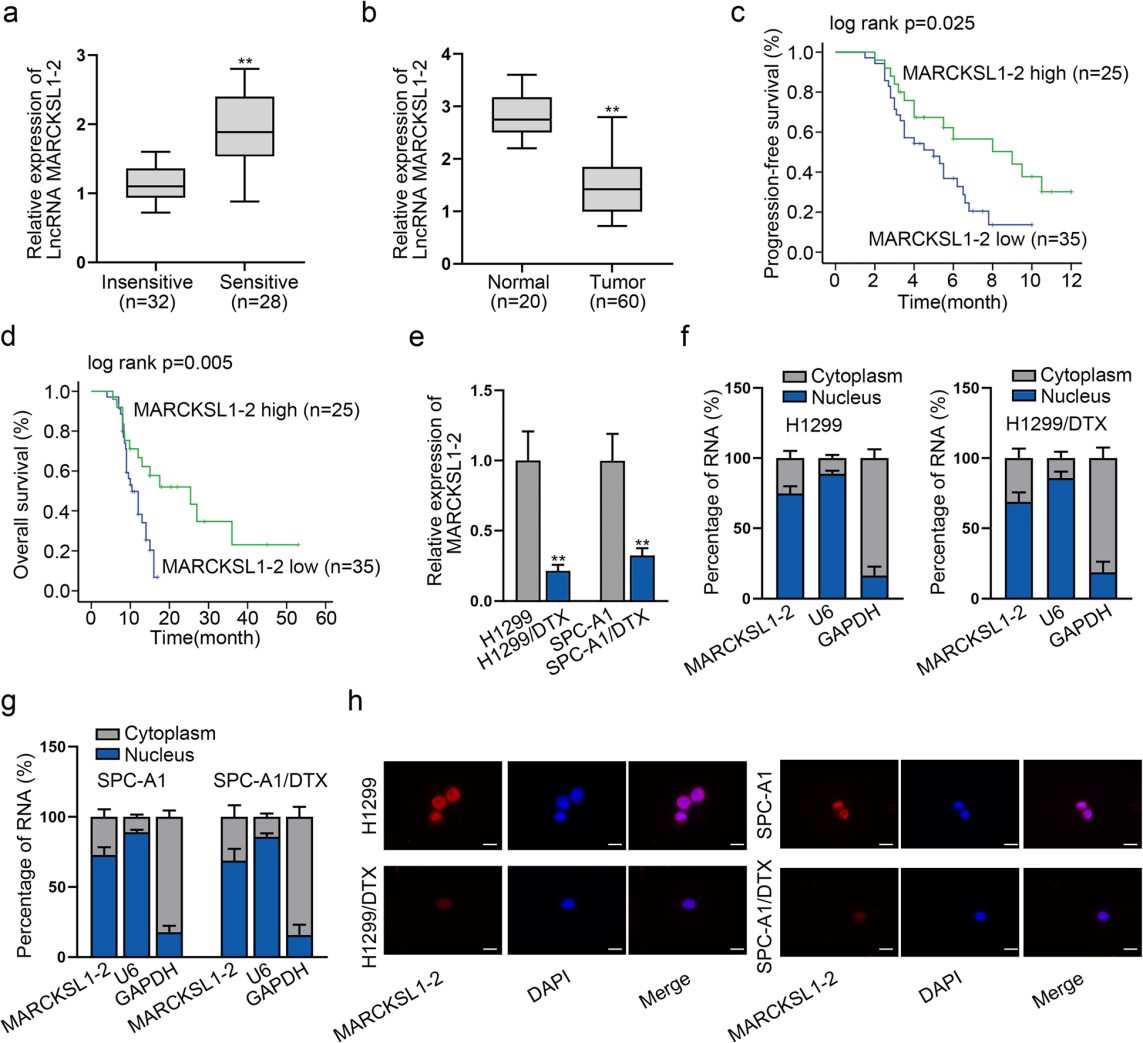
Down-regulation of lncRNA MARCKSL1–2 is associated with DTX resistance and poor prognosis in LAD patients. **a** MARCKSL1–2 expression was detected by RT-qPCR in the DTX sensitive- (CR + PR; *n* = 28) and insensitive- (SD + PD; *n* = 32) LAD tissues. **b** MARCKSL1–2 expression in normal lung tissues (*n* = 20) and LAD tissues (*n* = 60) was determined by RT-qPCR. **c** Kaplan-Meier analysis of the association between progression-free survival (PFS) and MARCKSL1–2 level in LAD patients. **d** Kaplan-Meier analysis of the association between overall survival (OS) and MARCKSL1–2 level in LAD patients. **e** RT-qPCR tested the expression of MARCKSL1–2 in DTX-resistant LAD cells relative to parental H1299 or SPC-A1 cells. **f**-**g** Subcellular fractionation analyzed MARCKSL1–2 distribution in LAD cells and matched DTX-resistant cells. **h** IF analysis of MARCKSL1–2 distribution in parental and DTX-resistant LAD cells. Scale bar = 20 μm. ^**^P < 0.01

According to the tumor response to chemotherapy, 60 LAD tissues were divided into DTX insensitive- (SD + PD; *n* = 32) and sensitive- (CR + PR; *n* = 28) groups. Interestingly, RT-qPCR data indicated that the level of lncRNA MARCKSL1–2 in the insensitive LAD tissues was remarkably down-regulated in comparison with that in the sensitive group (Fig. 1a). Moreover, the level of MARCKSL1–2 in tumor tissues was lower than that in normal lung tissues (Fig. 1b). Furthermore, low MARCKSL1–2 expression was associated with poor chemotherapy response, advanced clinical stage, and poor histological differentiation (Supplementary Table 1). Kaplan-Meier analysis results revealed that lower MARCKSL1–2 level was correlated with a shorter PFS and OS of LAD patients (Fig. 1c-d). Additionally, MARCKSL1–2 expression was identified as one of the independent prognostic factors of LAD patients by univariate and multivariate Cox regression models (Supplementary Table 2). Based on the above data, we evaluated the association of MARCKSL1–2 with DTX resistance in LAD cells. In this regard, we first tested the expression pattern of MARCKSL1–2 in parental human LAD cells (H1299 and SPC-A1) and the matched DTX-resistant cells (H1299/DTX and SPC-A1/DTX). RT-qPCR unveiled that the level of MARCKSL1–2 was significantly declined in the DTX-resistant LAD cells compared with parental cells (Fig. 1e), suggesting that MARCKSL1–2 is related to the DTX resistance of human LAD cells. Before exploring its function, we analyzed the cellular distribution of MARCKSL1–2 in parental and DTX-resistant LAD cells. The data exhibited that MARCKSL1–2 principally existed in the nucleus (Figs. 1f-g). Similarly, FISH images indicated that the staining of MARCKSL1–2 was mainly in the nucleus, and less MARCKSL1–2 signals were observed in resistant cells than in parental cells (Fig. 1h). These results displayed that MARCKSL1–2 is expressed at low level in DTX-resistant LAD cells .

### Effects of MARCKSL1–2 on the behaviors of DTX-resistant or sensitive LAD cells under DTX treatment

**Fig. 2:**
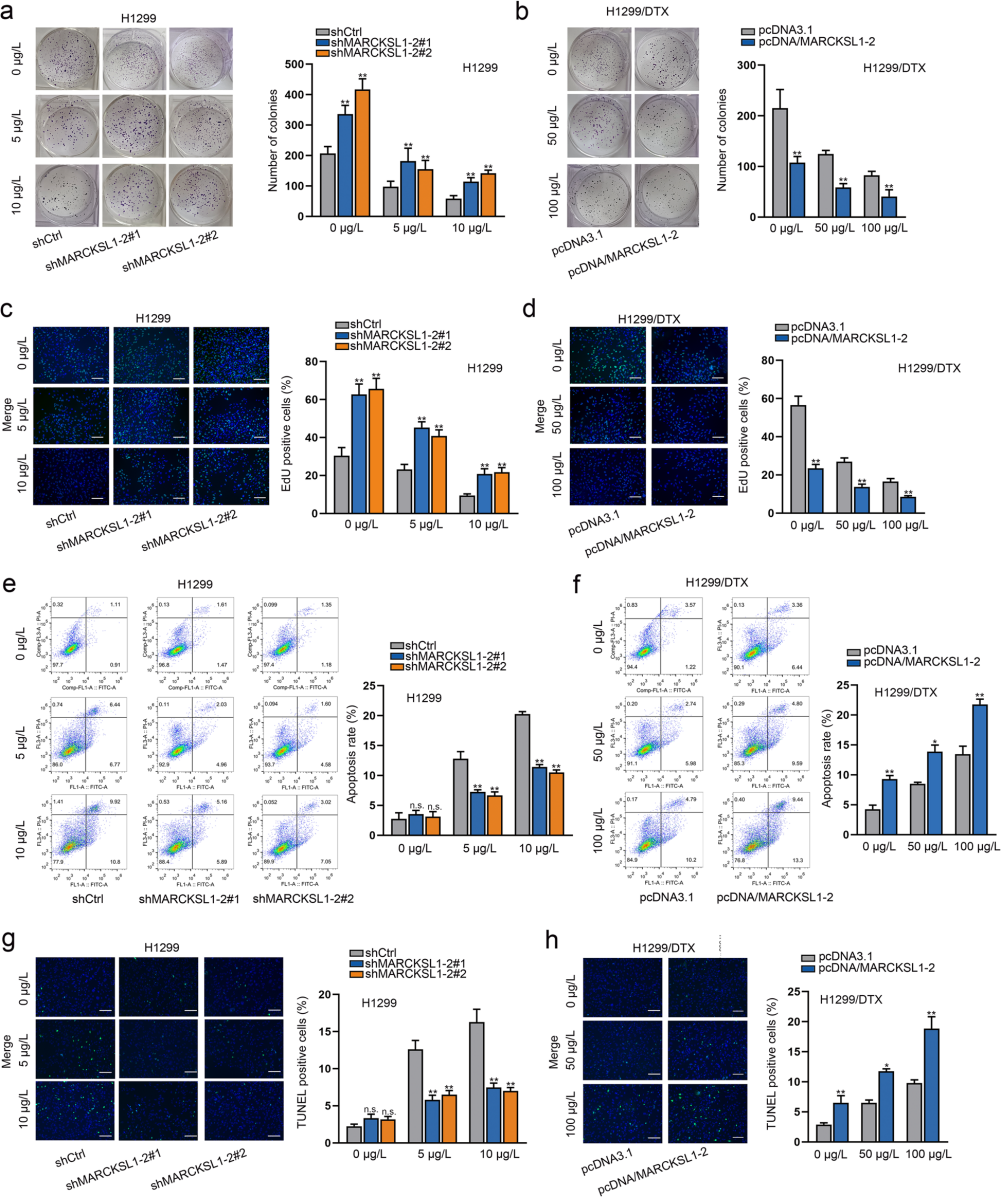
Effects of MARCKSL1–2 on the proliferation and apoptosis of DTX-sensitive or -resistant LAD cells under increasing dose of DTX treatment. H1299 cells were transfected with shRNAs targeting MARCKSL1–2, and H1299/DTX cells were transfected with pcDNA/MARCKSL1–2. **a**-**d** Colony formation and EdU assays (Scale bar = 100 μm) measured the impact of MARCKSL1–2 inhibition or overexpression on the proliferation capacity of H1299 cells treated with different doses of DTX (0, 5 and 10 μg/L) and H1299/DTX cells treated with different doses of DTX (0, 50 and 100 μg/L). **e**-**h** Flow cytometry analyses and TUNEL assays (Scale bar = 100 μm) detected the apoptosis rate of H1299 cells and H1299/DTX cells under different conditions. ^*^P < 0.05, ^**^P < 0.01. n.s.: no significance

To probe the relationship between MARCKSL1–2 and DTX resistance, we first analyzed its expression of MARCKSL1–2 in the parental and DTX-resistant LAD cells in response to increasing doses of DTX. As shown in Supplementary Fig. 1a-b, the level of MARCKSL1–2 was gradually increased in a dose-dependent manner in different LAD cells. To determine the biological function of MARCKSL1–2 in LAD cells, we silenced MARCKSL1–2 expression in H1299 and SPC-A1 cells while up-regulated MARCKSL1–2 expression in H1299/DTX and SPC-A1/DTX cells (Supplementary Fig. 1c-d). As such, the IC_50_ value of parental cells was increased after MARCKSL1–2 silencing, whereas that of DTX-resistant cells was decreased after MARCKSL1–2 overexpression (Supplementary Fig. 1e-f). Furthermore, the proliferation ability was obviously promoted when MARCKSL1–2 was silenced in H1299 and SPC-A1 cells. On the contrary, MARCKSL1–2 overexpression led to hampered proliferation of H1299/DTX and SPC-A1/DTX cells. Consistently, DTX treatment hindered the proliferation of different LAD cells in a dose-dependent manner (Figs. 2a-d, Supplementary Fig. 2a-d). Additionally, the results from flow cytometry analyses and TUNEL assays showed that MARCKSL1–2 deletion lessened the apoptotic rate of H1299 and SPC-A1 cells treated with 5 or 10 μg/L of DTX, whereas MARCKSL1–2 up-regulation induced the apoptosis of H1299/DTX and SPC-A1/DTX cells treated with 0, 50 or 100 μg/L DTX (Figs. 2e-h, Supplementary Fig. 2e- h). These results suggested that MARCKSL1–2 plays a growth-inhibitory role in both DTX-sensitive and -resistant LAD cells.

### MARCKSL1–2 mitigates DTX resistance in LAD cells by regulating HDAC1 and miR-200b

**Fig. 3:**
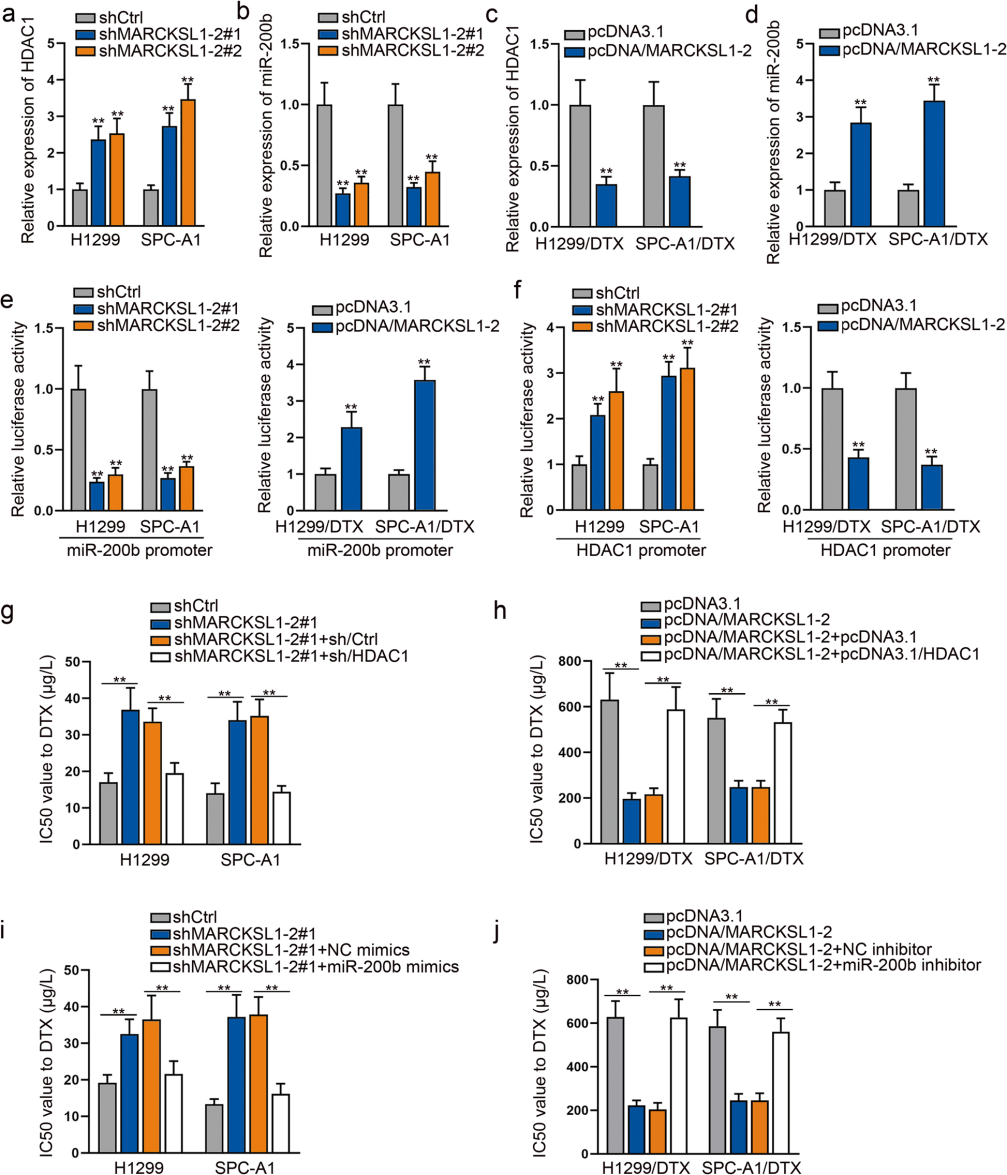
MARCKSL1–2 inhibits DTX resistance in LAD cells by regulating HDAC1 or miR-200b. **a**-**b**. HDAC1 and miR-200b expression was assessed using RT-qPCR in H1299 and SPC-A1 cells with or without MARCKSL1–2 interference. **c**-**d** By using RT-qPCR, the expression of HDAC1 or miR-200b was assessed in H1299/DTX and SPC-A1/DTX cells transfected with pcDNA3.1 or pcDNA/MARCKSL1–2. **e**-**f** Luciferase reporter assays evaluated the luciferase activity of miR-200b or HDAC1 promoter in LAD cells with MARCKSL1–2 depletion and in DTX-resistant LAD cells with MARCKSL1–2 overexpression. **g**-**h** The IC_50_ value of indicated LAD cells to DTX treatment was assessed by CCK-8 assays. **i**-**j** The IC_50_ value of DTX in indicated LAD cells was assessed by CCK-8 assays. ^**^P < 0.01

Our previous study indicated that HDAC1/4 and miR-200b are involved in DTX resistance of LAD cells [23]. Here, we investigated whether MARCKSL1–2 could regulate HDAC1/4 and miR-200 to reduce DTX resistance. Interestingly, we found that MARCKSL1–2 negatively regulated HDAC1 and positively modulated miR-200b in both parental and DTX-resistant LAD cells (Figs. 3a-d), whereas it had no apparent effect on the levels of HDAC4 and miR-200a/c (Supplementary Fig. 3). Because MARCKSL1–2 was mainly distributed in the nucleus of LAD cells, we evaluated the impacts of MARCKSL1–2 on the transcription of HDAC1 and miR-200b. Data from luciferase reporter assays showed that the luciferase intensity of miR-200b promoter was reduced by MARCKSL1–2 knockdown in H1299 and SPC-A1 cells, but increased when MARCKSL1–2 was up-regulated in H1299/DTX and SPC-A1/DTX cells (Fig. 3e). Conversely, the luciferase activity of HDAC1 promoter showed opposite trends to that of miR-200b promoter in indicated cells (Fig. 3f). More importantly, it was validated that the enhanced IC_50_ value to DTX induced by MARCKSL1–2 knockdown could be countervailed in response to HDAC1 interference or miR-200b mimics, and the reduced IC_50_ value to DTX mediated by MARCKSL1–2 overexpression was restored in face of HDAC1 overexpression or miR-200b inhibition (Fig. 3g-j). Taken together, MARCKSL1–2 attenuates DTX resistance in LAD cells by repressing HDAC1 or elevating miR-200b.

### MARCKSL1–2 recruits SUZ12 in LAD cells

**Fig. 4:**
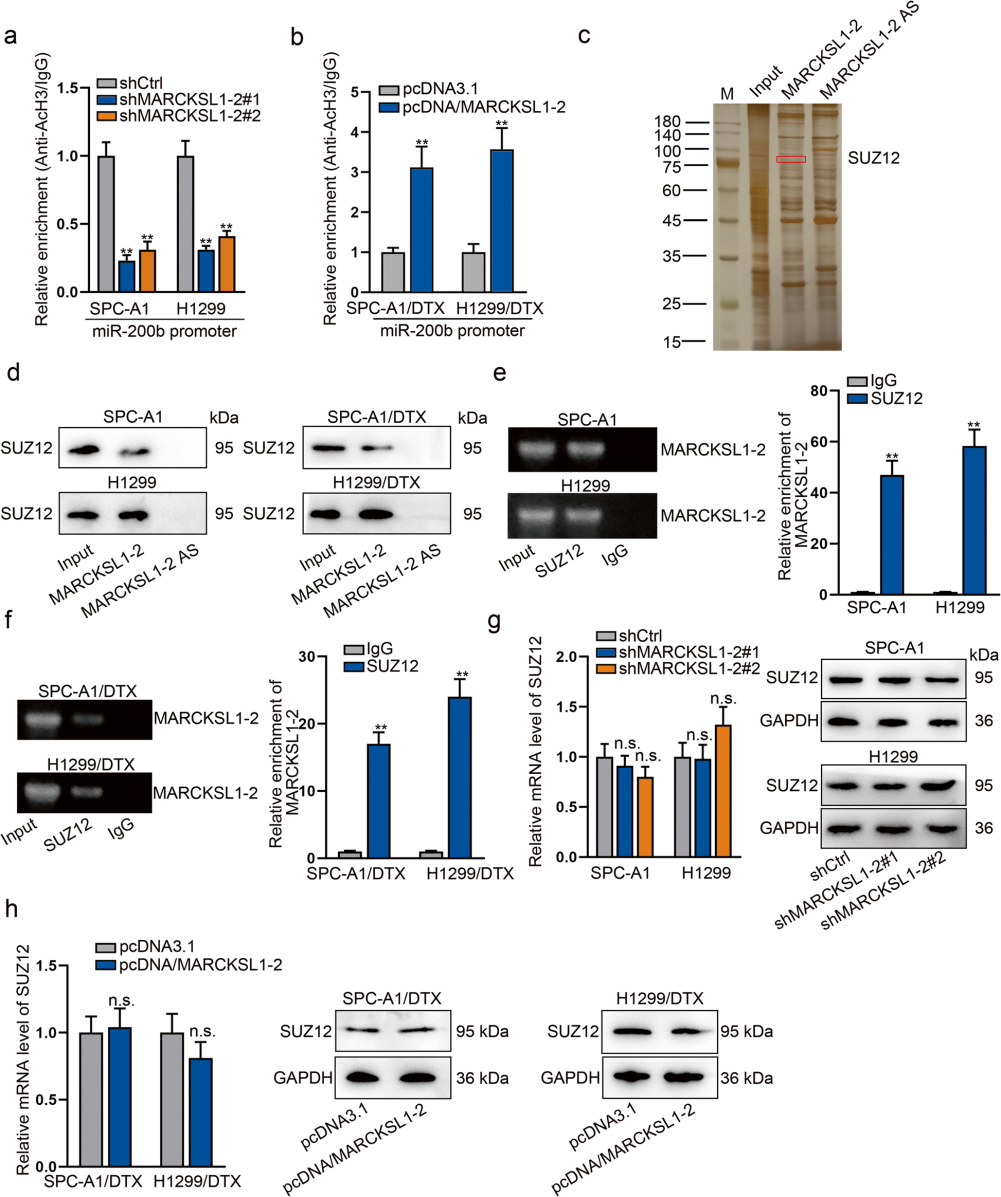
MARCKSL1–2 interacts with SUZ12 in LAD cells. **a**-**b** ChIP assay detected the changes in histone-H3 acetylation level at miR-200b promoter in SPC-A1/H1299 cells under shMARCKSL1–2 suppression and that in SPC-A1/DTX/H1299/DTX cells upon MARCKSL1–2 overexpression. **c** RNA pull-down assay followed by mass spectrometry analysis examined the proteins binding with MARCKSL1–2. **d** RNA pull- down plus Western blot detected the existence of SUZ12 in the pull-down complexes of MARCKSL1–2. **e**-**f** RIP assays followed by AGE or RT-qPCR examined the enrichment of MARCKSL1–2 in SUZ12 groups. g-h. RT-qPCR and Western blot detected the influence of MARCKSL1–2 on SUZ12 expression in indicated LAD cells. ^**^P < 0.01. n.s.: no significance

According to the previous report, HDAC1/4 deletion can elevate miR-200b expression by maintaining histone-H3 acetylation at miR-200b promoter and therefore reverse DTX resistance of LAD cells [14]. Based on this, we hypothesized that MARCKSL1–2 could inhibit HDAC1 to up-regulate miR-200b by enhancing histone-H3 acetylation level at miR-200b promoter. The ChIP analysis showed that MARCKSL1–2 reduction inhibited while its upregulation enhanced the histone-H3 acetylation level of miR-200b promoter (Fig. 4a-b), which supported our hypothesis. Next, we probed into the mechanism whereby MARCKSL1–2 affected HDAC1 transcription. Because lncRNAs modulate transcription mainly through binding to different proteins involved in transcriptional process [24], we employed RNA pull-down and silver staining to analyze the potential proteins interacting with MARCKSL1–2. After spectrometry analysis, we found that SUZ12 interacted with MARCKSL1–2 with a high binding score (Fig. 4c). Moreover, we confirmed the existence of SUZ12 in MARCKSL1–2 pulled down complexes in both parental and DTX-resistant LAD cells (Fig. 4d). Meanwhile, RIP data revealed that MARCKSL1–2 was highly enriched in anti-SUZ12 groups, and its enrichment in DTX-resistant LAD cells was higher than that in parental cells (Figs. 4e-f). Additionally, we detected the impacts of MARCKSL1–2 on the mRNA level and protein level of SUZ12. Results demonstrated that MARCKSL1–2 was not capable of affecting SUZ12 expression in different LAD cells (Figs. 4g-h). All these results revealed that MARCKSL1–2 interacts with SUZ12 in both parental and DTX- resistant LAD cells.

### MARCKSL1–2 affects HDAC1 expression by recruiting SUZ12 to modulate H3K27me3 level at HDAC1 promoter

**Fig. 5:**
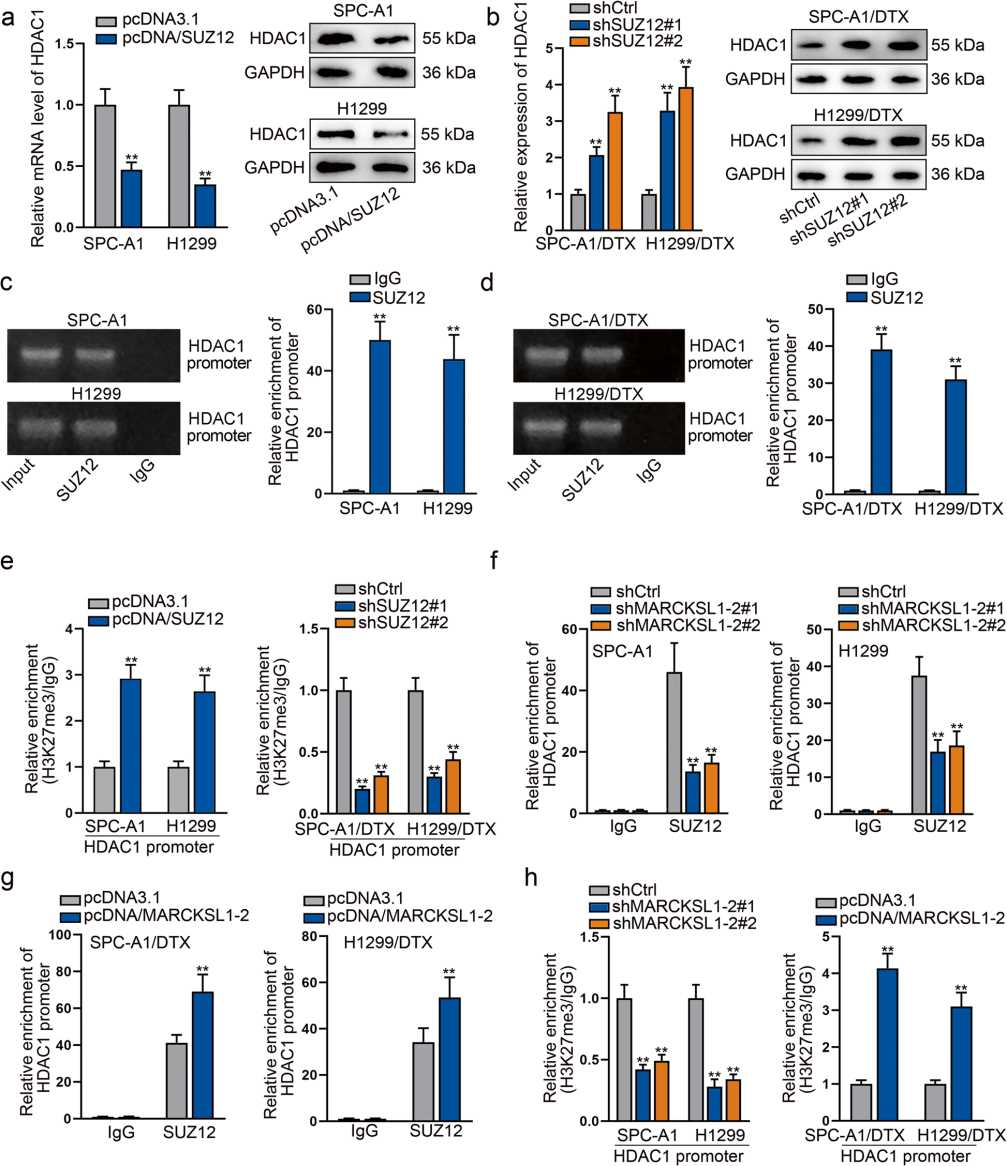
MARCKSL1–2 epigenetically inhibits HDAC1 expression by recruiting SUZ12 to HDAC1 promoter. **a**-**b** The impact of SUZ12 on the mRNA and protein levels of HDAC1 assessed by RT-qPCR and Western blot in indicated LAD cells. **c**-**d** ChIP assay plus AGE or RT-qPCR was used to evaluate the enrichment of HDAC1 promoter in SUZ12 groups. **e** ChIP assay analyzed the impact of SUZ12 on the H3K27me3 modification at HDAC1 promoter in indicated LAD cells. **f**-**g** ChIP assay was conducted to evaluate the changes in enrichment of HDAC1 promoter recognized by SUZ12 in SPC-A1/H1299 cells with MARCKSL1–2 inhibition and in DTX-resistant LAD cells with MARCKSL1–2 upregulation. **h** The influence of MARCKSL1–2 silence or overexpression on the level of H3K27me3 modification at HDAC1 promoter assessed by ChIP experiments. ^**^P < 0.01

SUZ12 is a key subunit of PRC2 complex which regulates the H3K27 methylation of target genes [25, 26]. Therefore, we further explored whether MARCKSL1–2 modulated HDAC1 transcription through its recruitment of SUZ12. We first analyzed the influence of SUZ12 on HDAC1. As expected, we found that SUZ12 up-regulation reduced the mRNA and protein levels of HDAC1 in SPC-A1 and H1299 cells, whereas loss of SUZ12 resulted in elevated levels of HDAC1 in H1299/DTX and SPC-A1/DTX cells (Supplementary Fig. 4a-b, Figs. 5a-b). Subsequently, ChIP assay showed that HDAC1 promoter was enriched in the SUZ12 groups of both parental and DTX-resistant LAD cells, and the enrichment of HDAC1 promoter in DTX-resistant cells was less than that in the parental cells (Figs. 5c-d). In addition, it was proved that the H3K27me3 level in HDAC1 promoter was enhanced in response to SUZ12 upregulation while lowered in face of SUZ12 downregulation (Fig. 5g). More interestingly, the enrichment of HDAC1 promoter in SUZ12 groups was evidently reduced by MARCKSL1–2 knockdown in SPC-A1 and H1299 cells, whereas strengthened when MARCKSL1–2 was up-regulated in SPC-A1/DTX and H1299/DTX cells (Figs. 5f-g). Additionally, the H3K27me3 level in HDAC1 promoter was decreased under MARCKSL1–2 interference but increased upon MARCKSL1–2 overexpression (Fig. 5h). These results revealed that MARCKSL1–2 hinders HDAC1 expression by recruiting SUZ12 to induce H3K27me3 modification at HDAC1 promoter.

### MARCKSL1–2 affects DTX resistance of LAD cells by regulating HDAC1/miR-200b axis

**Fig. 6:**
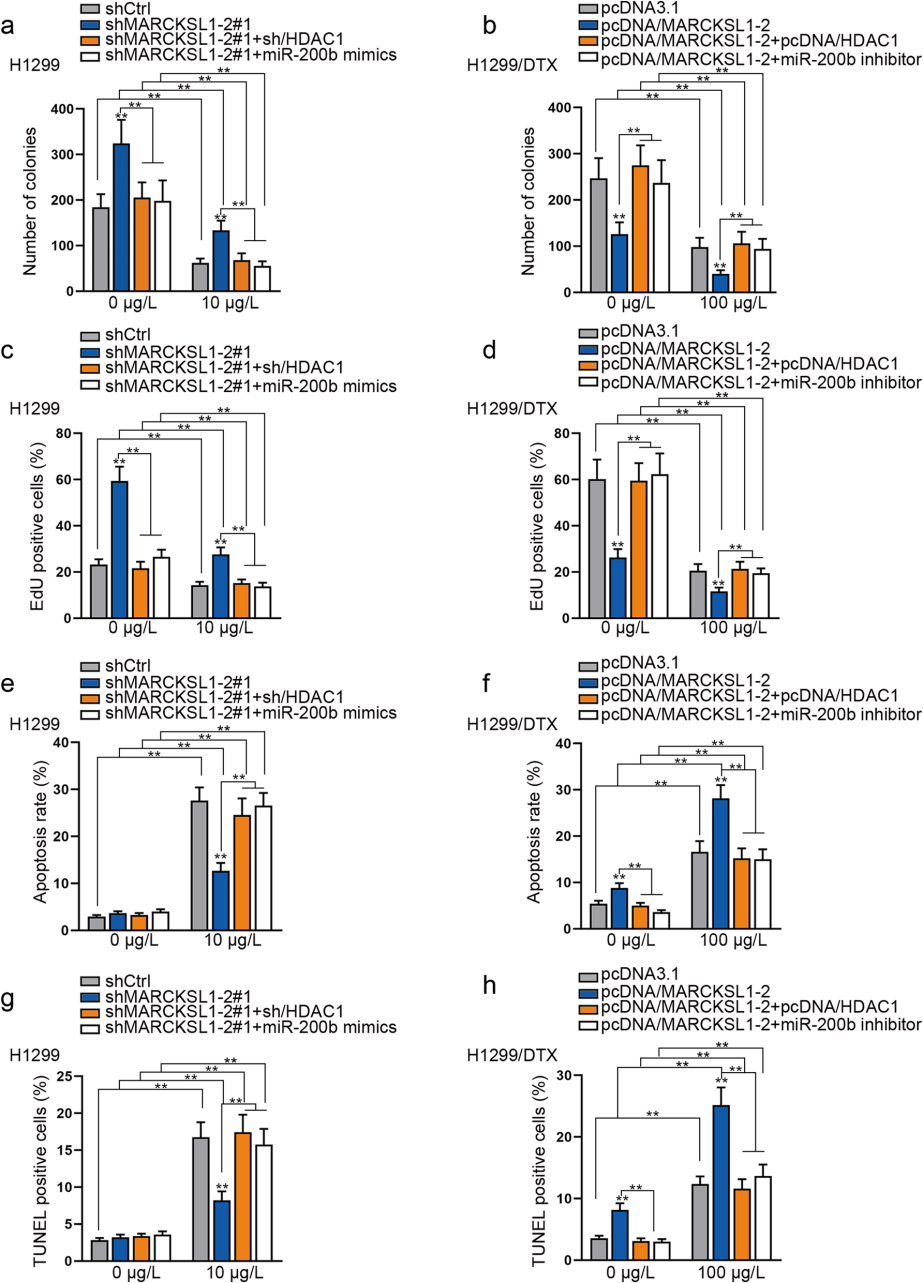
MARCKSL1–2 affects DTX resistance of LAD cells by regulating HDAC1/miR-200b axis. H1299 cells were transfected with shCtrl, shMARCKSL1–2#1, shMARCKSL1–2#1 + sh/HDAC1 or shMARCKSL1–2#1 + miR-200b mimics. H199/DTX cells were transfected with pcDNA3.1, pcDNA/MARCKSL1–2, pcDNA/MARCKSL1–2 + pcDNA/HDAC1 or pcDNA/MARCKSL1–2 + miR-200b inhibitor. Then, rescue assays were conducted on these groups of cells. a-d. Colony formation and EdU assays detected the proliferation of indicated H1299 and H1299/DTX cells. e-h. Flow cytometry analyses and TUNEL assays examined the apoptosis of indicated H1299 and H1299/DTX cells. ^**^P < 0.01. n.s.: no significance

Next, we tested whether MARCKSL1–2 modulates DTX resistance of LAD cells through its regulation on HDAC1 and miR-200b. We performed gain- and loss-of-function experiments. We demonstrated that the level of MARCKSL1–2 decreased by shMARCKSL1–2, but it remained unchanged after co-transfection with sh/HDAC1 or miR-200b mimics (Supplementary Fig. 4c); the reduced level of miR-200b caused by MARCKSL1–2 depletion was recovered by the inhibition of HDAC1 or overexpression of miR-200b (Supplementary Fig. 4d), and the elevated HDAC1 expression by MARCKSL1–2 silencing was only reversed by HDAC1 inhibition but not affected by miR-200b mimics (Supplementary Fig. 4e). In DTX-resistant cells, MARCKSL1 expression, which was enhanced by the transfection of MARCKSL1–2 overexpression vector, remained unchanged after HDAC1 overexpression or miR-200b inhibition (Supplementary Fig. 4f). The enhanced miR-200b expression by the up-regulation of MARCKSL1–2 was counteracted by the overexpression of HDAC1 or inhibition of miR-200b (Supplementary Fig. 4 g). Besides, the reduced HDAC1 level induced by MARCKSL1–2 overexpression was recovered by the transfection of its own overexpression vector but remained unchanged by miR-200b inhibition (Supplementary Fig. 4 h). Then, results from colony formation and EdU assays displayed that MARCKSL1–2 knockdown elevated proliferation ability of H1299 and SPC-A1 cells, and the elevated proliferation was drastically reversed under HDAC1 suppression or miR-200b mimics. Meanwhile, the proliferation ability of H1299/DTX and SPC-A1/DTX cells impeded by MARCKSL1–2 up-regulation was entirely restored in face of HDAC1 up-regulation or miR-200b inhibition (Figs. 6a-d, Supplementary Fig. 5a-d). Data of Flow cytometry and TUNEL assays showed that HDAC1 knockdown or miR-200b mimicking rescued the inhibitory effects on the apoptosis of LAD cells due to MARCKSL1–2 deletion. Reversely, HDAC1 overexpression or miR-200b inhibition fully abolished the enhancing effect of MARCKSL1–2 up-regulation on the apoptosis of DTX-resistant LAD cells (Figs. 6e-h, Supplementary Fig. 5e- h). These results suggested that MARCKSL1–2 eases the DTX resistance of LAD cells by regulating HDAC1/miR-200b axis.

### MARCKSL1–2 alleviates DTX resistance of LAD tumor in vivo

**Fig. 7:**
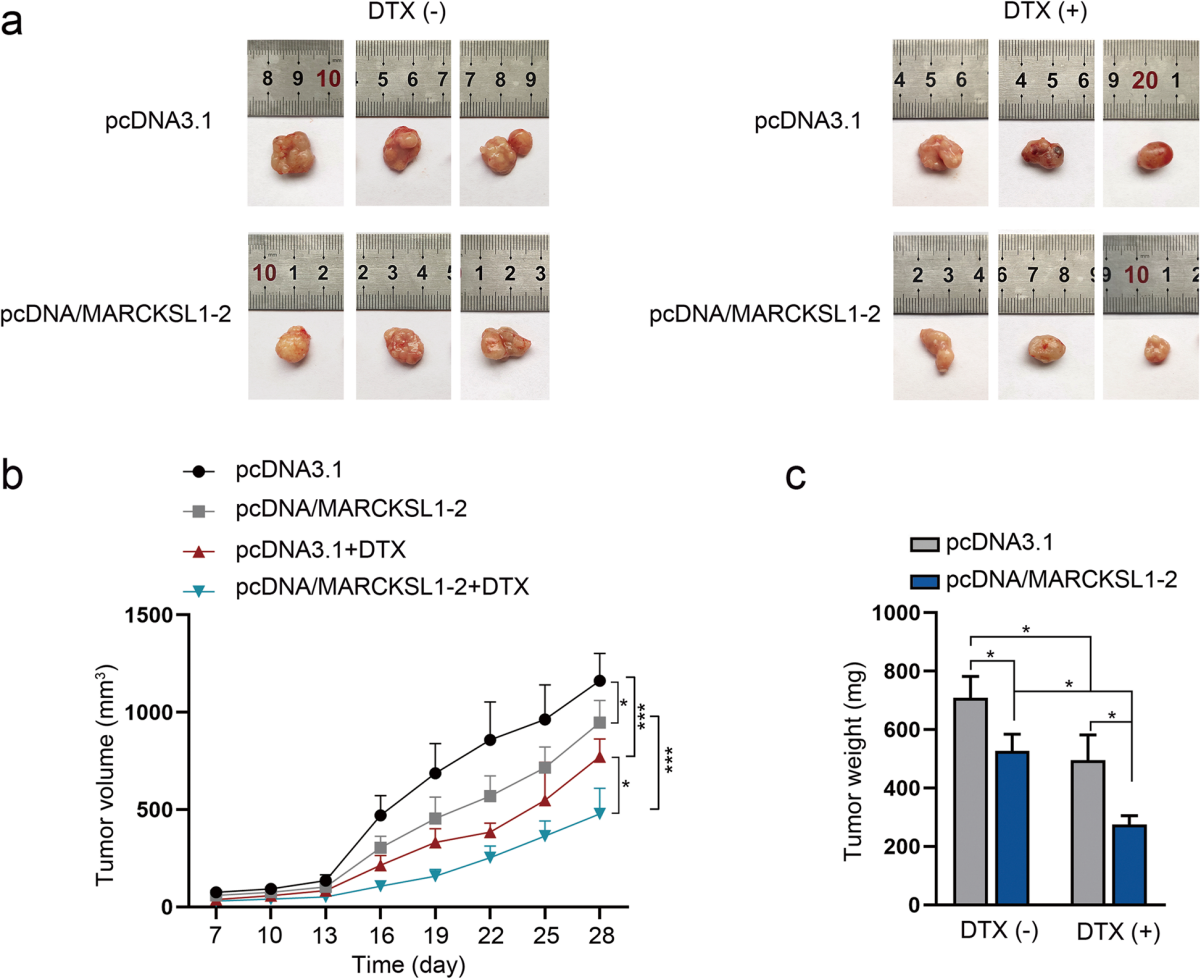
MARCKSL1–2 suppresses tumor growth and DTX resistance in vivo. **a** Tumors resected from mice in four different groups (pcDNA3.1 or pcDNA3.1/MARCKSL1–2-transfected H1299/DTX cells with or without DTX treatment). **b**-**c** Tumor volume and weight in four different groups. ^*^P < 0.05, ^***^P < 0.001

To further validate the significance of MARCKSL1–2 in DTX resistance in vivo, we constructed mouse models and observed the tumor growth in mice with different treatments. It was observed that tumor size, volume, and weight were smaller in the group with MARCKSL1–2 overexpression or DTX treatment than those in the control group, and MARCKSL1–2 overexpression induced further reduction in the above parameters in groups with DTX treatment (Figs. 7a-c). Therefore, we confirmed that MARCKSL1–2 enhances the sensitivity of LAD cells to DTX treatment in vivo.

## Discussion

We uncovered that lncRNA MARCKSL1–2 was expressed at a low level in DTX-resistant LAD cells, and it assuaged the DTX-resistance and LAD cell proliferation, while elevated the apoptosis of LAD cells. Moreover, MARCKSL1–2 up-regulated miR-200b to reverse DTX resistance of LAD cells by recruiting SUZ12 to suppress HDAC1 level.

Increasing evidence supported that lncRNAs can function in the DTX resistance of LAD cells, suggesting that they could be a novel potential target for LAD treatment. For instance, Jing Chen et al. found that CCAT1 elevates chemoresistance and promotes epithelial-to-mesenchymal transition process in DTX-resistant LAD cells [27]. Moreover, linc-ROR facilitates chemoresistance in DTX-resistant LAD cells by targeting miR-145 to regulate FSCN1 [28]. Herein, we investigated the role of a novel lncRNA MARCKSL1–2 in the DTX resistance of LAD. Our study found that MARCKSL1–2 was expressed at a low level in LAD tissues compared with adjacent normal tissues. Moreover, the level of MARCKSL1–2 in DTX-insensitive LAD patient tissues was lower than that in DTX-sensitive LAD patient tissues. In addition, we found that low level of MARCKSL1–2 was associated with the poor prognosis of LAD patients, confirming the prognostic significance of MARCKSL1–2 in LAD patients. Functionally, we revealed that MARCKSL1–2 worked as an inhibitor of the DTX resistance and growth of LAD cells.

Recently, epigenetic regulation has been broadly reported in the development of drug resistance in cancer cells [29–31]. Accordingly, molecules targeting epigenetic regulators have been emerged as a new strategy for cancer treatment [30–32]. Our previous finding unveiled that HDAC1/4 epigenetically regulated miR-200b to enhance DTX resistance in human LAD cells [14]. Interestingly, the present study confirmed that MARCKSL1–2 could affect the expression of HDAC1 and miR-200b in both parental and DTX-resistant LAD cells. MiR-200b was suggested as a potential biomarker in LAD [33], and it has been proved to be associated with DTX-resistance in LAD cells through regulating autophagy [34] or by targeting E2F3 [35]. Histone deacetylase 1 (HDAC1) is an enzyme with a function of removing the acetyl group from lysine residue, and mainly acts as an epigenetic regulator that regulates gene transcription [36]. Besides our former findings [14], the epigenetic inhibition of HDAC1 on miR-200b has also been validated in cancer stem-like cells [23]. Consistent with the previous findings, here we found that MARCKSL1–2 up-regulated the expression of miR-200b by inhibiting HDAC1 to enhance the histone-H3 acetylation at miR-200b promoter.

Furthermore, we found that MARCKSL1–2 recruited SUZ12 to induce H3K27me3 modification at HDAC1 promoter, therefore repressing HDAC1 transcription and expression in LAD cells. SUZ12 is a member of the polycomb repressive complex 2 (PRC2) which induces gene repression-related H3K27me3 at target gene promoter in many cancers [37–39]. Previous studies have shown that up-regulation of H3K27me3 can sensitize osteosarcoma to cisplatin [40]. In our present study, we demonstrated that the presence of MARCKSL1–2 reinforced the binding of SUZ12 to HDAC1 promoter to enhance H3K27me3 level at HDAC1 promoter, resulting in reduced HDAC1 expression and therefore elevating miR-200b level in LAD cells. Additionally, in vivo study indicated that MARCKSL1–2 overexpression hindered tumor growth and strengthened the efficacy of DTX treatment in vivo. Current literatures have uncovered the relationship of HDAC1 or miR-200b with the resistance of cancer cells to several commonly used drugs in LAD, such as cisplatin, paclitaxel, and gefitinib [41–46]. These findings suggested the potential involvement of MARCKSL1–2/HDAC1/miR-200b axis in the acquired resistance of LAD cells to multiple drugs.

There are some limitations in our current study. For example, the upstream and downstream regulating molecules of MARCKSL1–2 remained unclear and required further investigation. Besides, whether MARCKSL1–2 plays a role in the development of resistance to other drugs in LAD cells needs further validations in future studies.

## Conclusions

Our research elucidated the inhibitory role of MARCKSL1–2/SUZ12/HDAC1/miR-200b axis in the growth and DTX resistance of LAD cells, suggesting that MARCKSL1–2 could be a promising target to improve the chemotherapy of LAD patients.

## Supplementary Information

**Additional file 1: Supplementary Table 1.** Correlations between LncRNA MARCKSL1–2 and clinicopathological characteristics of LAD patients. **Supplementary Table 2.** The analysis of prognostic variables by Cox regression model.

**Additional file 2: Supplementary Fig. 1.** MARCKSL1–2 expression is associated with DTX resistance in LAD cells. a-b. The level of MARCKSL1–2 was examined by RT-qPCR in the parental and DTX-resistant LAD cells treated with increasing doses of DTX. c-d. The inhibition or overexpression efficiencies of MARCKSL1–2 was detected by RT-qPCR in parental or DTX-resistant LAD cells. e-f. The IC_50_ value of LAD cells to DTX treatment was estimated by CCK-8 assay in parental LAD cells under MARCKSL1–2 interference and SPC-A1/DTX and H1299/DTX cells under MARCKSL1–2 upregulation. ^*^P < 0.05, ^**^P < 0.01.

**Additional file 3: Supplementary Fig. 2.** Effects of MARCKSL1–2 on the growth and DTX-resistance of LAD cells. SPC-A1 cells were transfected with shRNAs targeting MARCKSL1–2, and SPC-A1/DTX cells were transfected with pcDNA/MARCKSL1–2. a-d. Colony formation and EdU assays (Scale bar = 100 μm) measured the proliferation ability of SPC-A1 cells treated with DTX (0, 5, 10 μg/L) and SPC-A1/DTX cells treated with DTX (0, 50, 100 μg/L). e-h. Flow cytometry analyses and TUNEL assays (Scale bar = 100 μm) detected the apoptosis of SPC-A1 cells treated with DTX (0, 5, 10 μg/L) and SPC-A1/DTX cells treated with DTX (0, 50, 100 μg/L). ^*^P < 0.05, ^**^P < 0.01. n.s.: no significance.

**Additional file 4: Supplementary Fig. 3.** Effect of MARCKSL1–2 on the expression of HDAC4, miR-200a and miR-200c. a. The expression of HDAC4 was examined by RT-qPCR in parental LAD cells with or without MARCKSL1–2 silencing. b. The level of miR-200a/c was tested by RT-qPCR in parental LAD cells with or without MARCKSL1–2 silencing. c. The level of HDAC4 was detected by RT-qPCR in DTX-resistant LAD cells in response to MARCKSL1–2 overexpression. d. The level of miR-200a/c was analyzed by RT-qPCR in DTX-resistant LAD cells with or without MARCKSL1–2 overexpression. n.s.: no significance.

**Additional file 5: Supplementary Fig. 4.** Expression changes of SUZ12, MARCKSL1-AS1, miR-200b and HDAC1 in indicated parental and DTX-resistant LAD cells. a-b. RT-qPCR and Western blot examined the overexpression efficiency of SUZ12 in H1299 and SPC-A1 cells and the interference efficiency of SUZ12 in SPC-A1/DTX and H1299/DTX cells. c-e. The levels of MARCKSL1–2, miR-200b and HDAC1 in LAD cells transfected with shCtrl, shMARCKSL1–2#1, shMARCKSL1–2#1 + sh/HDAC1 or shMARCKSL1–2#1 + miR-200b mimics. f-h. The levels of MARCKSL1–2, miR-200b and HDAC1 in DTX-resistant cells transfected with pcDNA3.1, pcDNA/MARCKSL1–2, pcDNA/MARCKSL1–2 + pcDNA/HDAC1 or pcDNA/MARCKSL1–2 + miR-200b inhibitor. ^**^P < 0.01. n.s.: no significance.

**Additional file 6: Supplementary Fig. 5.** MARCKSL1–2 affects the growth and DTX-resistance of LAD cells through regulating HDAC1 or miR-200b. SPC-A1 cells were transfected with shCtrl, shMARCKSL1–2#1, shMARCKSL1–2#1 + sh/HDAC1 or shMARCKSL1–2#1 + miR-200b mimics. SPC-A1/DTX cells were transfected with pcDNA3.1, pcDNA/MARCKSL1–2, pcDNA/MARCKSL1–2 + pcDNA/HDAC1 or pcDNA/MARCKSL1–2 + miR-200b inhibitor. Then rescue assays were conducted in these groups of cells. a-d. Colony formation and EdU assays detected the proliferation ability of indicated SPC-A1 and SPC-A1/DTX cells. e-h. Flow cytometry analyses and TUNEL assays examined the apoptosis of indicated SPC-A1 and SPC-A1/DTX cells. ^**^P < 0.01.

## Data Availability

All data and supporting materials are available upon request by writing to the corresponding authors.

## Abbreviations

NSCLC: Non-small cell lung cancer
LAD: Lung adenocarcinoma
DTX: Docetaxel
LncRNA: Long non-coding RNA
MARCKSL1–2: MARCKS like 1-transcript variant 2
miR-200b: MicroRNA-200b
H3K27me3: Histone H3 lysine 27 trimethylation
HDAC1: Histone deacetylase 1
SUZ12: Suppressor of zeste 12
RT-qPCR: Quantitative real-time polymerase chain reaction
FISH: Fluorescent in situ hybridization
EdU: 5-Ethynyl-2′-deoxyuridine
TUNEL: Terminal-deoxynucleoitidyl Transferase Mediated Nick End labeling
CCK-8: Cell counting kit-8
ChIP: Chromatin immunoprecipitation
RIP: RNA immunoprecipitation
PD: Progressive disease
SD: Stable disease
CR: Complete response
PR: Partial response
AS: Antisense
PFS: Progression-free survival
OS: Overall survival
