# Supplementary material for: MARCKSL1–2 reverses docetaxel-resistance of lung adenocarcinoma cells by recruiting SUZ12 to suppress HDAC1 and elevate miR-200b
Source: Mol Cancer. 2022 Jul 21;21:150. doi: 10.1186/s12943-022-01605-w (PMC9306054; doi:10.1186/s12943-022-01605-w)
Supplement: Supplementary file 1 — Additional file 1: Supplementary Table 1. Correlations between LncRNA MARCKSL1–2 and clinicopathological characteristics of LAD patients. Supplementary Table 2. The analysis of prognostic variables by Cox regression model. [file 12943_2022_1605_MOESM1_ESM.docx]

**Supplementary table 1. Correlations between LncRNA MARCKSL1-2 and clinicopathological characteristics of LAD patients**

| Variables | *n* | LncRNA MARCKSL1-2 expression | | *P*-value |
| --- | --- | --- | --- | --- |
|  |  | High (n=25) | Low (n=35) |  |
| Age (years) |  |  |  | 0.793 |
| <60 | 30 | 13 | 17 |  |
| ≥60 | 30 | 12 | 18 |  |
| Gender |  |  |  | 0.275 |
| Female | 29 | 10 | 19 |  |
| Male | 31 | 15 | 16 |  |
| Clinical stage |  |  |  | 0. 004** |
| IIIB | 32 | 19 | 13 |  |
| IV | 28 | 6 | 22 |  |
| Chemotherapy response |  |  |  | 0.005** |
| CR+PR | 28 | 17 | 11 |  |
| SD+PD | 32 | 8 | 24 |  |
| Differentiation |  |  |  | 0.015* |
| Well+Moderate | 25 | 15 | 10 |  |
| Poor | 35 | 10 | 25 |  |

Lung adenocarcinoma: LAD; complete or partial response: CR+PR; stable or progressive disease: SD+PD. **P*<0.05, ***P*<0.01.

**Supplementary table 2. The analysis of prognostic variables by Cox regression model**

| **Variables** | **Univariate analysis** | ***P*-value** | **Multivariate analysis** | ***P*-valu**e |
| --- | --- | --- | --- | --- |
|  | **RR (95%CI)** |  | **RR (95%CI)** |  |
| Clinical stage | 1.85 1.29-2.13 | 0.034* | 1.68 1.14-1.93 | 0.040* |
| (IIIB/IV) |  | | |  |
| ECOG | 2.17 1.45-2.65 | 0.025* | 1.73 1.56-2.17 | 0.035* |
| (0-1/>1) |  | | |  |
| MARCKSL1-2 expression | 0.63 0.35-0.88 | 0.010* | 0.71 0.42-0.92 | 0.022* |
| (Low/High) |  | | |  |
| HDAC1 expression | 2.91 1.79-3.46 | 0.020* | 2.10 1.84-3.68 | 0.038* |
| (Low /High) |  | | |  |

RR: relative ratio; MARCKSL1-2: LncRNA MARCKSL1-2; 95% CI: 95% confidence intervals; ECOG: Eastern Cooperative Oncology Group. **P*<0.05.
