# Supplementary figures and images for: MARCKSL1–2 reverses docetaxel-resistance of lung adenocarcinoma cells by recruiting SUZ12 to suppress HDAC1 and elevate miR-200b
Source: Mol Cancer. 2022 Jul 21;21:150. doi: 10.1186/s12943-022-01605-w (PMC9306054; doi:10.1186/s12943-022-01605-w)

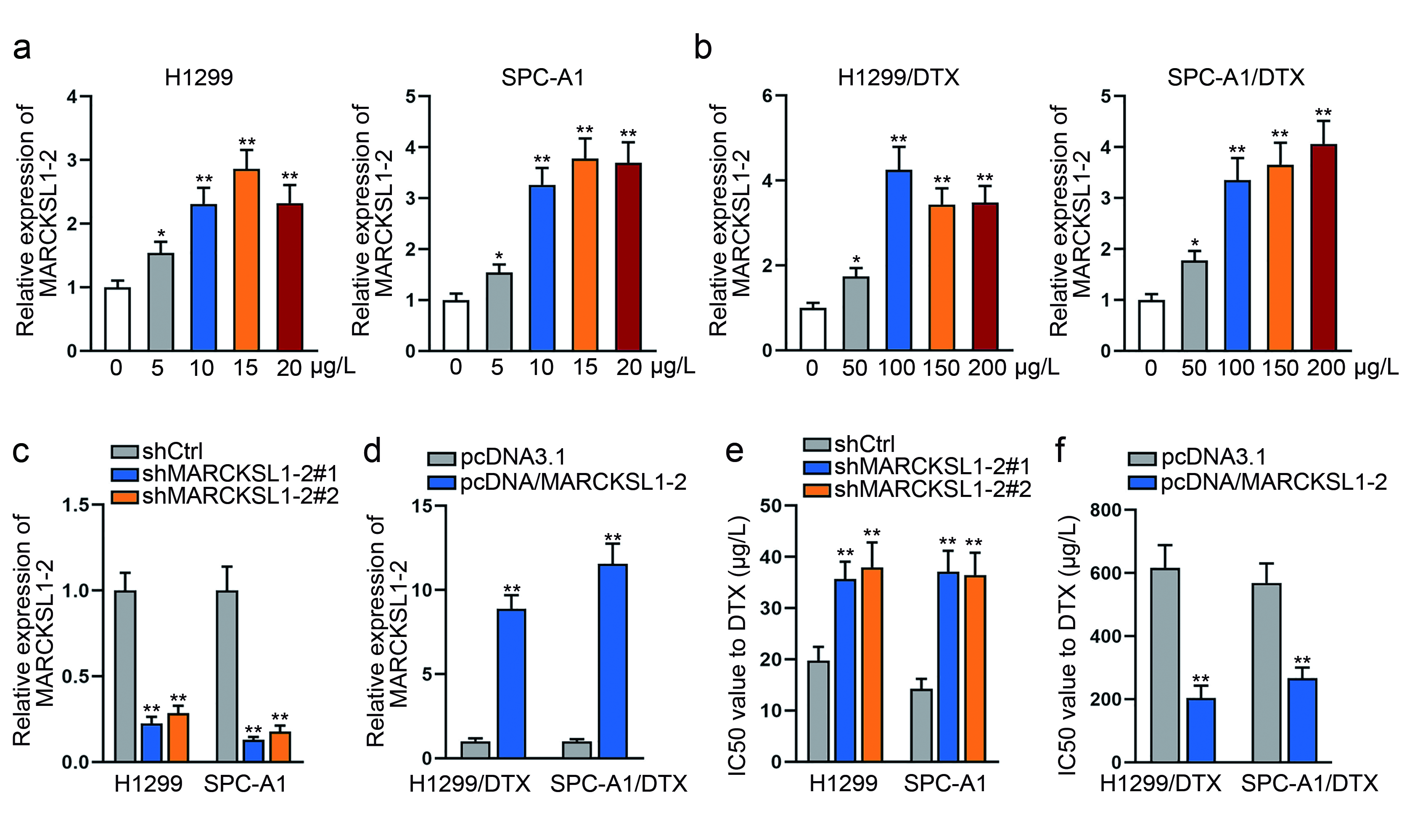

Supplement: Supplementary file 2 — Additional file 2: Supplementary Fig. 1. MARCKSL1–2 expression is associated with DTX resistance in LAD cells. a-b. The level of MARCKSL1–2 was examined by RT-qPCR in the parental and DTX-resistant LAD cells treated with increasing doses of DTX. c-d. The inhibition or overexpression efficiencies of MARCKSL1–2 was detected by RT-qPCR in parental or DTX-resistant LAD cells. e-f. The IC50 value of LAD cells to DTX treatment was estimated by CCK-8 assay in parental LAD cells under MARCKSL1–2 interference and SPC-A1/DTX and H1299/DTX cells under MARCKSL1–2 upregulation. *P < 0.05, **P < 0.01. [file 12943_2022_1605_MOESM2_ESM.tif]

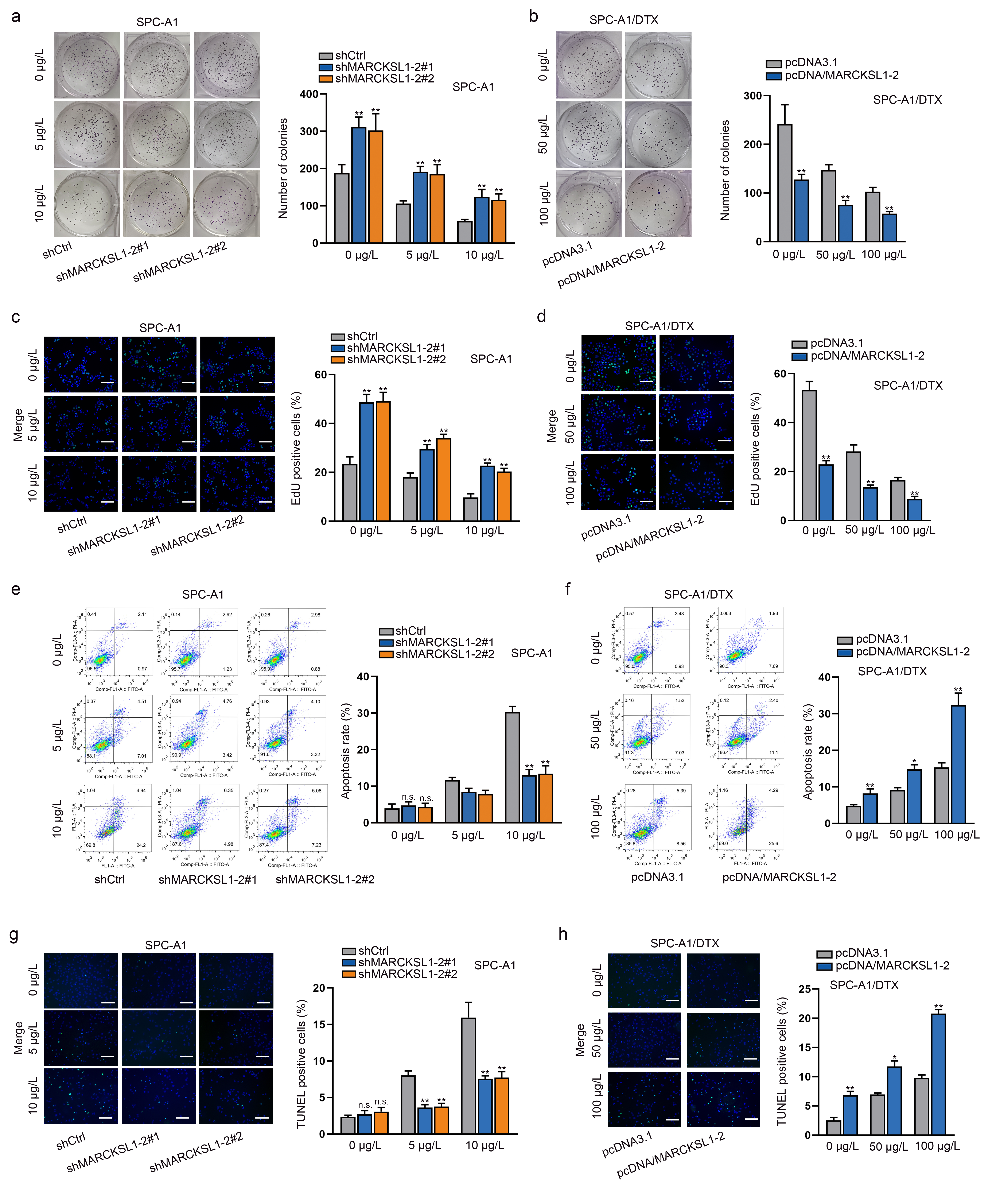

Supplement: Supplementary file 3 — Additional file 3: Supplementary Fig. 2. Effects of MARCKSL1–2 on the growth and DTX-resistance of LAD cells. SPC-A1 cells were transfected with shRNAs targeting MARCKSL1–2, and SPC-A1/DTX cells were transfected with pcDNA/MARCKSL1–2. a-d. Colony formation and EdU assays (Scale bar = 100 μm) measured the proliferation ability of SPC-A1 cells treated with DTX (0, 5, 10 μg/L) and SPC-A1/DTX cells treated with DTX (0, 50, 100 μg/L). e-h. Flow cytometry analyses and TUNEL assays (Scale bar = 100 μm) detected the apoptosis of SPC-A1 cells treated with DTX (0, 5, 10 μg/L) and SPC-A1/DTX cells treated with DTX (0, 50, 100 μg/L). *P < 0.05, **P < 0.01. n.s.: no significance. [file 12943_2022_1605_MOESM3_ESM.tif]

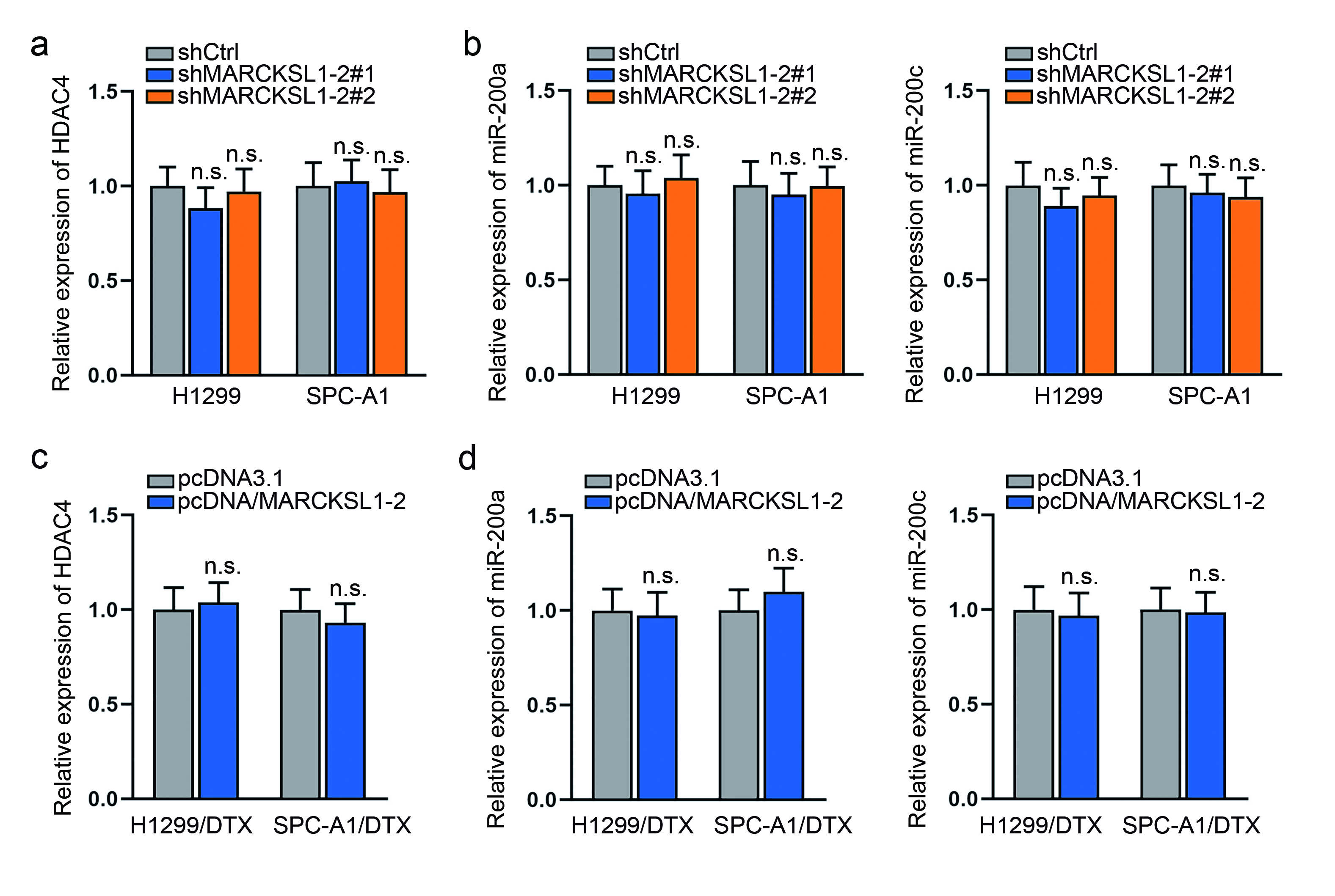

Supplement: Supplementary file 4 — Additional file 4: Supplementary Fig. 3. Effect of MARCKSL1–2 on the expression of HDAC4, miR-200a and miR-200c. a. The expression of HDAC4 was examined by RT-qPCR in parental LAD cells with or without MARCKSL1–2 silencing. b. The level of miR-200a/c was tested by RT-qPCR in parental LAD cells with or without MARCKSL1–2 silencing. c. The level of HDAC4 was detected by RT-qPCR in DTX-resistant LAD cells in response to MARCKSL1–2 overexpression. d. The level of miR-200a/c was analyzed by RT-qPCR in DTX-resistant LAD cells with or without MARCKSL1–2 overexpression. n.s.: no significance. [file 12943_2022_1605_MOESM4_ESM.tif]

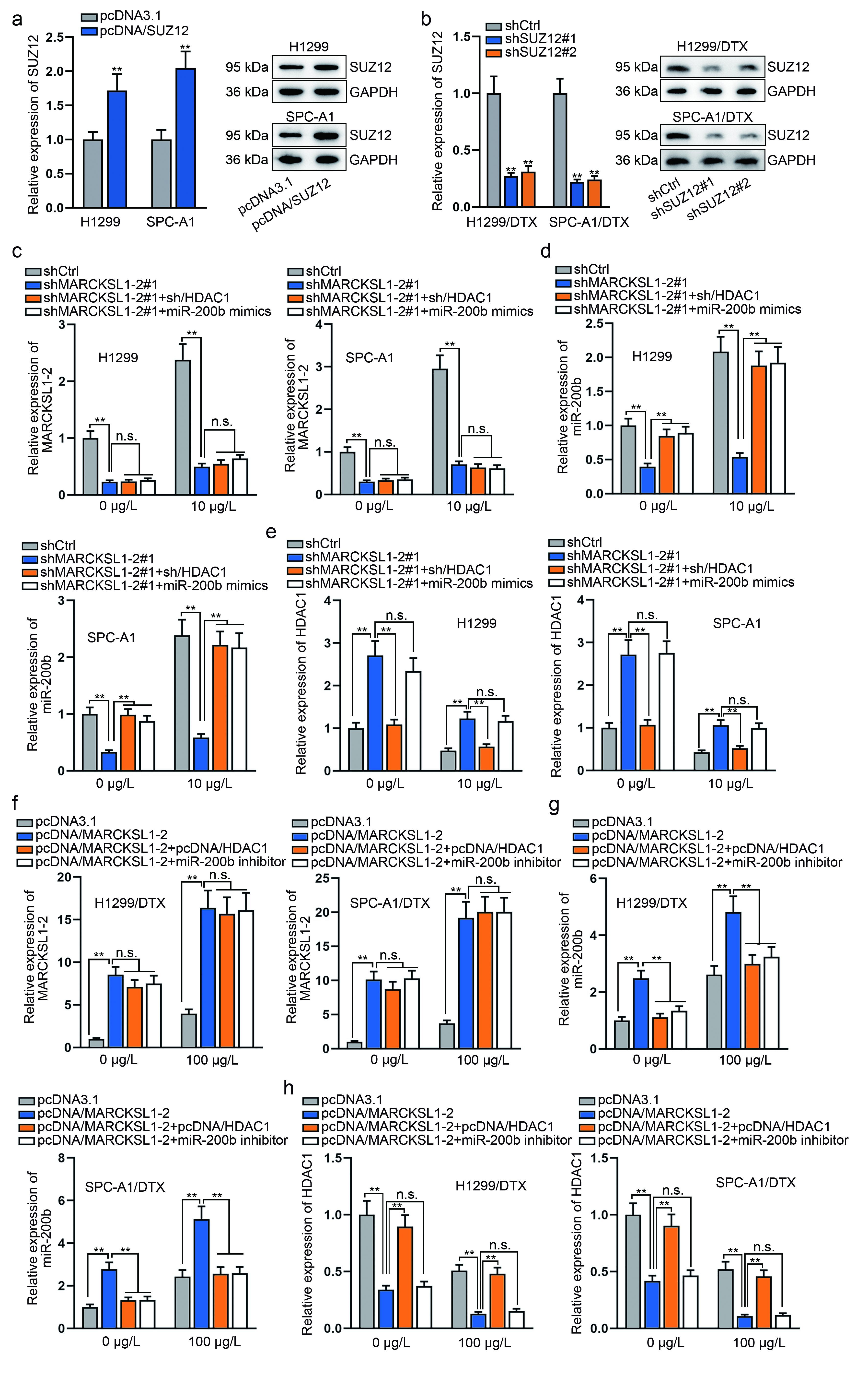

Supplement: Supplementary file 5 — Additional file 5: Supplementary Fig. 4. Expression changes of SUZ12, MARCKSL1-AS1, miR-200b and HDAC1 in indicated parental and DTX-resistant LAD cells. a-b. RT-qPCR and Western blot examined the overexpression efficiency of SUZ12 in H1299 and SPC-A1 cells and the interference efficiency of SUZ12 in SPC-A1/DTX and H1299/DTX cells. c-e. The levels of MARCKSL1–2, miR-200b and HDAC1 in LAD cells transfected with shCtrl, shMARCKSL1–2#1, shMARCKSL1–2#1 + sh/HDAC1 or shMARCKSL1–2#1 + miR-200b mimics. f-h. The levels of MARCKSL1–2, miR-200b and HDAC1 in DTX-resistant cells transfected with pcDNA3.1, pcDNA/MARCKSL1–2, pcDNA/MARCKSL1–2 + pcDNA/HDAC1 or pcDNA/MARCKSL1–2 + miR-200b inhibitor. **P < 0.01. n.s.: no significance. [file 12943_2022_1605_MOESM5_ESM.tif]

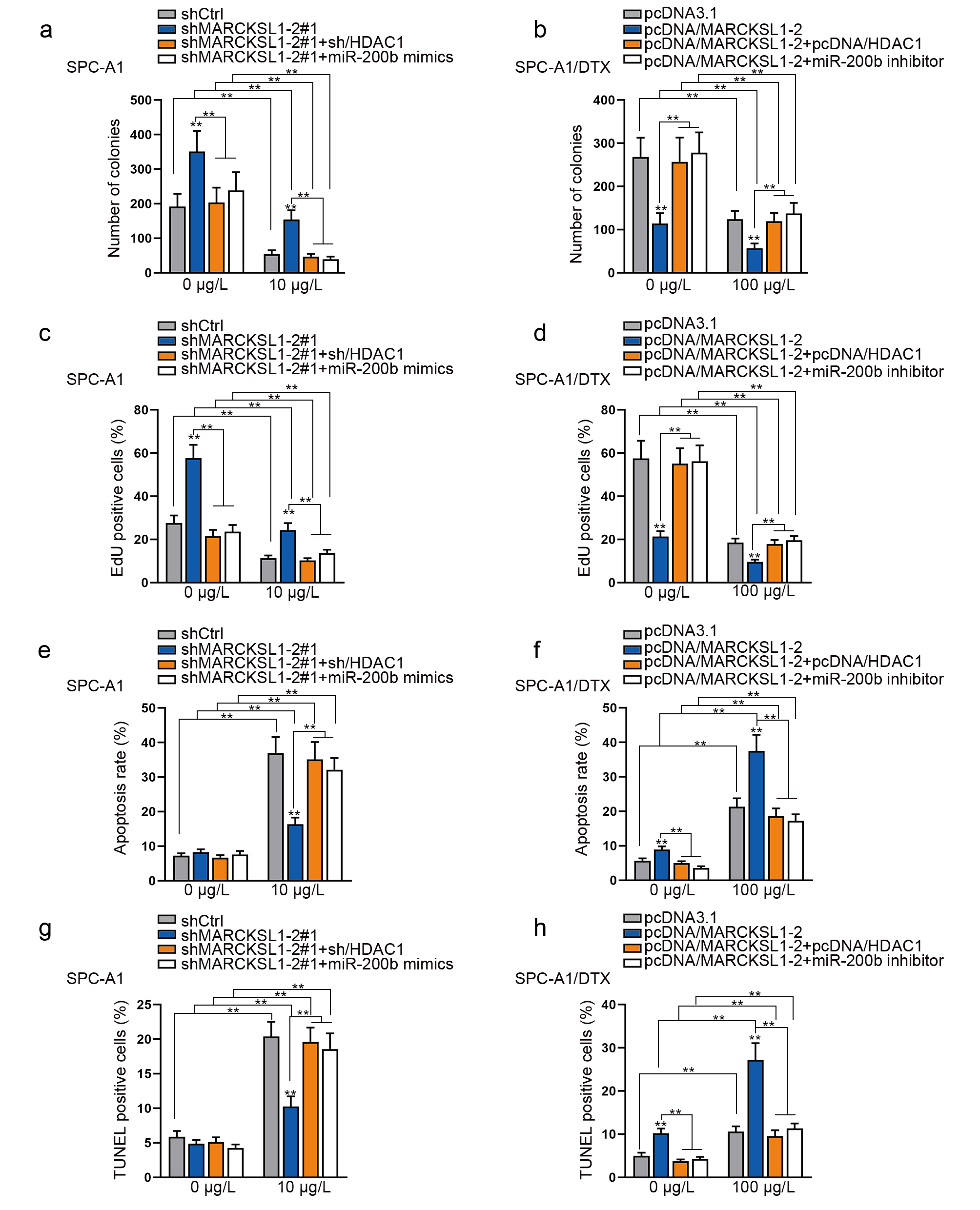

Supplement: Supplementary file 6 — Additional file 6: Supplementary Fig. 5. MARCKSL1–2 affects the growth and DTX-resistance of LAD cells through regulating HDAC1 or miR-200b. SPC-A1 cells were transfected with shCtrl, shMARCKSL1–2#1, shMARCKSL1–2#1 + sh/HDAC1 or shMARCKSL1–2#1 + miR-200b mimics. SPC-A1/DTX cells were transfected with pcDNA3.1, pcDNA/MARCKSL1–2, pcDNA/MARCKSL1–2 + pcDNA/HDAC1 or pcDNA/MARCKSL1–2 + miR-200b inhibitor. Then rescue assays were conducted in these groups of cells. a-d. Colony formation and EdU assays detected the proliferation ability of indicated SPC-A1 and SPC-A1/DTX cells. e-h. Flow cytometry analyses and TUNEL assays examined the apoptosis of indicated SPC-A1 and SPC-A1/DTX cells. **P < 0.01. [file 12943_2022_1605_MOESM6_ESM.tif]
